# A proof-of-concept study of the in-vivo validation of a computational fluid dynamics model of personalized radioembolization

**DOI:** 10.1038/s41598-021-83414-7

**Published:** 2021-02-16

**Authors:** Raúl Antón, Javier Antoñana, Jorge Aramburu, Ana Ezponda, Elena Prieto, Asier Andonegui, Julio Ortega, Isabel Vivas, Lidia Sancho, Bruno Sangro, José Ignacio Bilbao, Macarena Rodríguez-Fraile

**Affiliations:** 1 Universidad de Navarra, TECNUN Escuela de Ingeniería, 20018 Donostia-San Sebastián, Spain; 2 IdiSNA, Instituto de Investigación Sanitaria de Navarra, 31008 Pamplona, Spain; 3 Department of Radiology, Clínica Universidad de Navarra, 31008 Pamplona, Spain; 4 Department of Nuclear Medicine, Clínica Universidad de Navarra, 31008 Pamplona, Spain; 5 Department of Nuclear Medicine, Clínica Universidad de Navarra, 28027 Madrid, Spain; 6 Department of Hepatology, Clínica Universidad de Navarra, 31008 Pamplona, Spain; 7 CIBEREHD, Centro de Investigación Biomédica en Red de Enfermedades Hepáticas Y Digestivas, 28029 Madrid, Spain; 8 Present Address: Pontificia Universidad Católica de Valparaíso, Valparaíso, Chile

**Keywords:** Liver cancer, Computational models

## Abstract

Radioembolization (RE) with yttrium-90 (^90^Y) microspheres, a transcatheter intraarterial therapy for patients with liver cancer, can be modeled computationally. The purpose of this work was to correlate the results obtained with this methodology using in vivo data, so that this computational tool could be used for the optimization of the RE procedure. The hepatic artery three-dimensional (3D) hemodynamics and microsphere distribution during RE were modeled for six ^90^Y-loaded microsphere infusions in three patients with hepatocellular carcinoma using a commercially available computational fluid dynamics (CFD) software package. The model was built based on in vivo data acquired during the pretreatment stage. The results of the simulations were compared with the in vivo distribution assessed by ^90^Y PET/CT. Specifically, the microsphere distribution predicted was compared with the actual ^90^Y activity per liver segment with a commercially available 3D-voxel dosimetry software (PLANET Dose, DOSIsoft). The average difference between the CFD-based and the PET/CT-based activity distribution was 2.36 percentage points for Patient 1, 3.51 percentage points for Patient 2 and 2.02 percentage points for Patient 3. These results suggest that CFD simulations may help to predict ^90^Y-microsphere distribution after RE and could be used to optimize the RE procedure on a patient-specific basis.

## Introduction

Radioembolization (RE) with yttrium-90 (^90^Y) microspheres is a transcatheter intraarterial therapy that has emerged as a safe and effective treatment option for patients with primary or secondary liver cancer, such as hepatocellular carcinoma (HCC)^1^. RE consists of the intraarterial infusion of ^90^Y-loaded microspheres that are transported to the tumoral bed, where they emit tumor-killing doses of radiation^2^. Currently, a simulation of the RE procedure is performed with the intraarterial administration of ^99m^Tc-Macroaggregated of Albumin (^99m^Tc-MAA) on the assumption that the distribution of ^99m^Tc-MAA and ^90^Y-microspheres are similar. Nevertheless, systematic errors (e.g. differences in catheter position, injection techniques) or hemodynamic changes during both procedures can result in a poor correlation between ^99m^Tc-MAA and ^90^Y-microspheres distribution^3^. There have been in vitro studies where the density, flow dynamics and the embolization effect of several types of particles are experimentally analyzed^4–6^.

Three-dimensional (3D) simulation of hemodynamics and microsphere transport using computational fluid dynamics (CFD) has been designed to improve RE. These in silico (performed on computer) simulations allow the flow patterns of a fluid and the trajectories of some particles (in this case microspheres) to be predicted in a certain region of space, or computational domain, over time. This in silico approach has been successfully evaluated experimentally using a scaled model of a generalized hepatic artery^7^. Numerous investigations based on CFD models have analyzed the influence that local 3D parameter modifications (e.g., type of catheter, catheter-tip location, microsphere infusion velocity, etc.) have on the final distribution of the microspheres^8–14^. This information may contribute to enhance antitumor efficacy and minimize complications due to radiation of nontarget tissues. These simulations could also complement the conventional planning with ^99m^Tc-MAA by defining the ideal injection speed and catheter placement in standard scenarios. If the information provided by CFD models could be calculated for individual patients, it would enable the development of more efficient and personalized RE procedures. However, the results from CFD simulations have not yet been compared to the actual microsphere distribution in real patients to assess to what extent the model represents the real-life hepatic artery hemodynamics during RE.

In RE, ^90^Y PET/CT is commonly used to assess microsphere distribution. This imaging modality has been shown to be a reliable tool to assess activity deposition^15^, to accurately quantify the total activity delivered^16^ and to estimate the absorbed doses^17^.

The aim of this study was to assess the ability of personalized CFD models to predict the microsphere distribution observed in the ^90^Y PET/CT study performed in each patient after the RE procedure. The average activity (estimation of the number of ^90^Y-microspheres) that the computational simulation study predicted would reach the liver irrigated by the artery in which the ^90^Y-microspheres were injected was compared with the real average activity obtained in the ^90^Y PET/CT study after RE.

## Materials and methods

The University of Navarra ethics committee approved the protocol (186/2018) for this study and it was performed in accordance with the ethical standards laid down in the 1964 Declaration of Helsinki and all subsequent revisions. Informed consent was signed by each patient.

### Patient-specific model development

#### RE work-out

All patients were scanned on a third-generation dual-source CT (SOMATOM Force, Siemens Healthineers, Forchheim Germany) prior to the RE procedure. To analyze arterial liver perfusion^18,19^, sequential CT scanning of the hepatic volume was performed over time. Perfusion CT (pCT) consisted of a pre-contrast image, followed by dynamic image acquisitions during and after intravenous administration of the iodinated contrast agent. The pCT was also used for the patient-specific 3D geometry of the liver (i.e., tumor and non-tumor volumes, the hepatic arteries, the portal and hepatic veins, and the corresponding territories) using an automatic liver segmentation software package (MeVis Medical Solutions AG, Bremen, Germany). Additionally, dynamic CT images were processed on an external workstation equipped with commercially available CT perfusion software (“CT Body Perfusion”, Syngo.via, Siemens Healthineers, Erlangen, Germany) to obtain the arterial blood flow rate that irrigates the tumoral and the healthy tissue^20^ of each segment, parameters needed for building the computational simulation.

After being considered as candidates for RE by a hepatobiliary multidisciplinary team (MDT), on the same day all patients received planning and treatment as explained in Ezponda et al^21.^ During the RE procedure, SIR-Spheres resin microspheres (Sirtex Medical Europe Gmbh, Bonn, Germany) were administered and some parameters related to the infusion of the ^90^Y-microspheres, such as the precise location of the catheter, injection velocity and volume infused, were prospectively collected. The exact position of the catheter was captured in a planar angiographic image acquired during the RE procedure and later visually placed in the 3D MeVis images with the help of the interventional radiologist that administered the treatment. The injection velocity was obtained from video recordings of the RE procedure, considering also the geometric information of the syringe and the microcatheter. Total volume was the sum of the SIR-spheres delivery-vial (V-vial) and Glucose 5% solution volumes infused during RE.

The morning after the RE treatment, a ^90^Y PET/CT scan centred on the liver region (two beds, 10 min/bed) was performed using a Biograph mCT-TrueV (Siemens Healthineers, Erlangen, Germany). The reconstruction protocol used for ^90^Y was previously optimized^16^.

#### Methodology of the simulation

**Figure 1:**
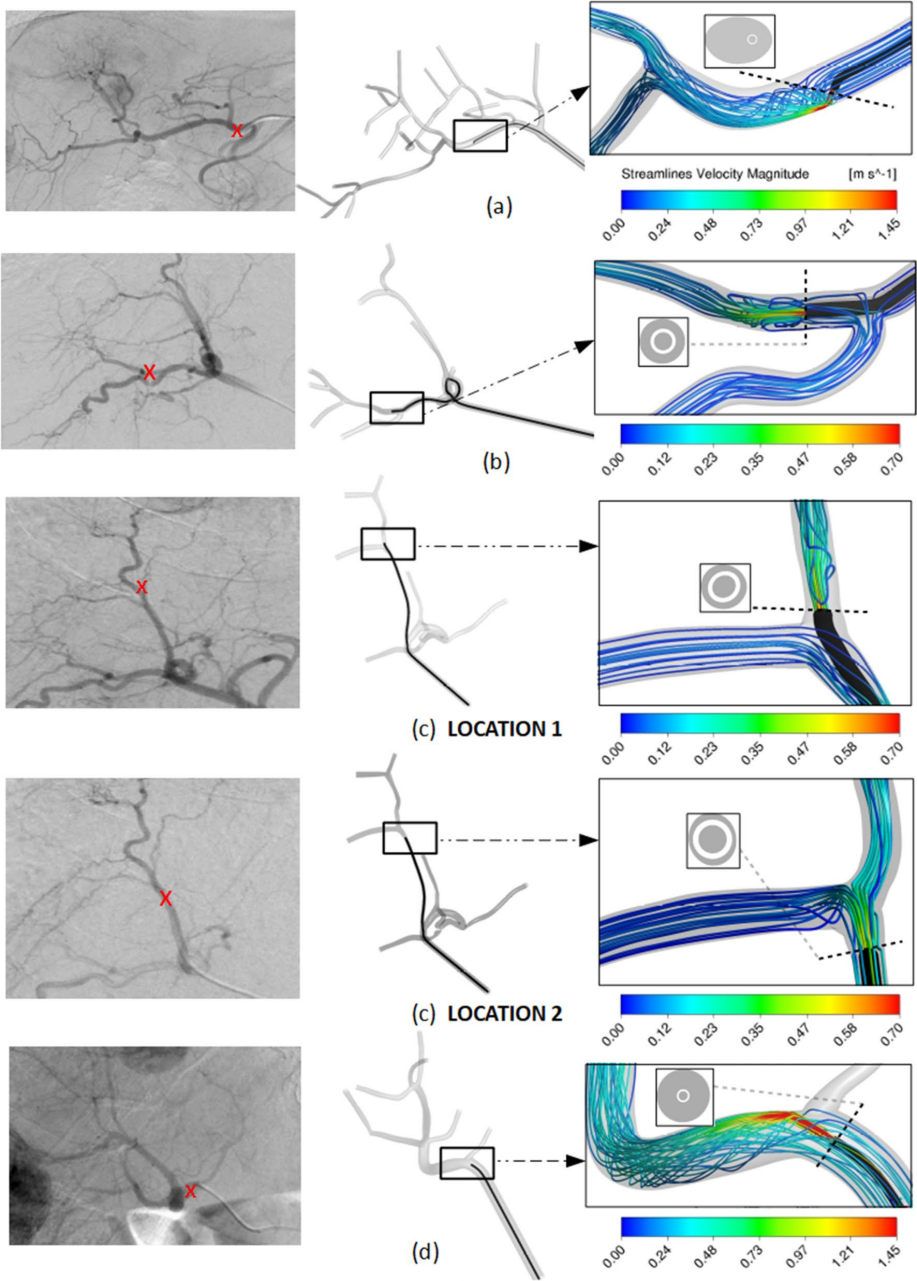
Visualization of microcatheter in the arteriography with the tip location indicated by a red cross (left) and in the CAD model (right) for (**a**) Patient 1, (**b**) Patient 2 and injection for tumors in segment 7, (**c**) Patient 2 and injection for tumors in segment 8, and (**d**) Patient 3. For each case, a detail of the CFD simulation results are shown in terms of flow streamlines colored by velocity magnitude, together with the microcatheter-tip position in the arterial lumen cross-section.

As previously mentioned, the CFD model predicts the fluid flow pressure, velocity and microsphere trajectory at any point, both temporal and spatial (computational domain) for the 3D geometry studied. CFD models are based on three main components: (1) the computational domain, (2) the boundary conditions (BCs) and (3) the equations governing the fluid-flow phenomena. (1) The computational domain is the part of the hepatic arterial tree analyzed (an artery branches successively giving rise to a tree-shaped pattern). In this study this included from the hepatic artery where the catheter was located up to the sub-segmental branches. The definition of the arterial boundaries of the domain is called truncation of the arterial tree. This truncation generates arterial inlets and outlets of the domain. To develop the computational domain, the MeVis liver segmentation computer-aided design (CAD) files containing the geometrical domain (hepatic artery) were used. This liver segmentation study also provided the vascular supply of the different liver segments and their volumetry (both healthy and tumor tissue). The catheter position was also included in the computational model (see Fig. 1) using SpaceClaim (ANSYS Inc., Canonsburg, PA, USA). (2) The BCs refer to the coupling of the hemodynamic conditions both inside and outside the computational domain. Therefore, there was one artery inlet and several domain outlets, each one supplying a different liver segment. Ideally, the BC should be measured in vivo. However, the most common approach is to model the flow circulation outside the computational domain^10^. For this model, the following parameters are needed: (a) volumes (ml) of healthy and tumoral tissue in each segment; (b) arterial perfusion (ml/min/ml) for healthy and tumor tissue in each segment, and (c) the segments irrigated by each of the outlets of the computational domain as is explained in detail in^22^. In this study, (a) and (c) were obtained from MeVis liver segmentation. In order to define (b), the following equation developed^22^ was used:

1 $$q_{s} = V_{0,s} k_{1,s}^{*} + V_{{{\text{c}},s}} k_{2,s} ,{ }\forall s \in \left\{ {{\text{S}}1, \ldots ,{\text{S}}8} \right\}$$

where $q_{s}$ is the arterial blood flow rate (ml/min) that feeds segment s, $V_{0,s}$ is the normal tissue volume and $V_{{{\text{c}},s}}$ is the tumor tissue volume contained in segment s (ml) (see Table 1), and $k_{1,s}^{*}$ and $k_{2,s}$ are the normal and tumor tissue arterial perfusions, both specific for segment s (ml/min/ml). These two perfusions ($k_{1,s}^{*}$ and $k_{2,s}$) were calculated in the pCT study. S1 to S8 correspond to segment 1 to segment 8.

**Table 1:** Tumoral and healthy volume distribution for each of the three patients.

Patient	Segment	Tumoral volume (ml)	Healthy volume (ml)
Patient 1	Segment 1	0.0	62.0
	Segment 2	0.8	127.2
	Segment 3	10.8	170.2
	Segment 4a	0.0	73.0
	Segment 4b	0.0	11.0
	Segment 5	0.0	124.0
	Segment 6	0.0	169.0
	Segment 7	23.4	349.6
	Segment 8	4.0	200.0
Patient 2	Segment 1	0.0	0.0
	Segment 2	0.0	119.7
	Segment 3	0.0	213.6
	Segment 4	0.0	70.0
	Segment 5	0.0	130.7
	Segment 6	0.0	119.0
	Segment 7	2.5	327.3
	Segment 8	4.5	156.8
Patient 3	Segment 1	0.0	16.0
	Segment 2	0.0	204.0
	Segment 3	0.0	188.0
	Segment 4	0.0	0.0
	Segment 5	30.7	122.3
	Segment 6	0.0	82.0
	Segment 7	38.1	389.9
	Segment 8	10.2	219.8

Depending on the number of outlets that irrigate one same segment, it is then assigned the healthy and tumor tissue volumes contained in that segment that each outlet irrigates. When assigning the healthy volume to each outlet, we assume that all the outlets will irrigate the same amount of healthy tissue within the segment, which means that the healthy volume irrigated by one outlet will be the total healthy volume divided by the number of outlets that irrigate that segment. For the tumor volume assignment, we rely on a manual process where we take into account the proximity of the relative position of the tumor to each outlet and then determine if the tumor volume is irrigated equally by the outlets or not.

The blood flow leaving each outlet is the segment’s healthy volume assigned to that outlet multiplied by the healthy perfusion for that segment, plus the tumor volume assigned to that outlet multiplied by the tumor’s perfusion of that segment. If a tumor is contained in x segments, we consider it as x tumors that are contained in each of those x segments.

The BC at the inlet is defined by the arterial flow rate that is the sum of the arterial flow rates of all the outlets. The boundary condition at the inlet of the microcatheter is defined by its injection velocity which was obtained from video recordings of the RE procedure for each patient. In this modeling approach, in order to reduce the time spent, only four cardiac cycles were simulated. ^90^Y-microspheres injected during the first cardiac cycle, and three additional cardiac cycles were modeled to ensure that most of the injected microspheres exit the computational domain. It assumes that the segment-to-segment microsphere distribution in the outlets of the computational domain during this period of time is representative of the whole procedure. With the simulation results, the segment-to-segment microsphere distribution is computed (it is beyond the scope of this model to account for the intrasegmental effects).

Once the geometry and the BCs are known, in order to obtain the fluid flow pressure, velocity and microsphere trajectory, it is necessary to solve 3) the equations governing the fluid (conservation of mass and conservation of linear momentum) and microsphere motion (Newton’s Second Law). For this purpose, the computational domain must be divided into millions of cells, a process called discretization of the domain. Nearby the catheter tip the mesh is fine enough to be able to resolve the jet that is formed at the microcatheter outlet. The discretization was performed using Fluent Meshing (ANSYS Inc.) as described in^10^. In this case, blood was assumed to be isothermal, incompressible, and non-Newtonian, and flowing in laminar regime, as in Aramburu et al.^10^. The gravity component was taken into consideration as 9.8 m/s^2^ in absolute value and the direction that corresponded to the patient in recumbent position. It is inside these cells where the equations are numerically solved by the commercial software (Fluent, ANSYS Inc. Canonsburg, PA, USA). The two-way coupled blood flow (continuous phase) and particle transport (discrete phase) models were solved. With regard to the equations governing the flow, the pressure–velocity coupling SIMPLE algorithm was used to solve the velocity and pressure in a segregated way. Spatial discretization was done by using the least squares cell-based algorithm for gradient computations and the second-order upwind scheme for the convection term of the momentum equations. For pressure interpolation, the second-order algorithm was employed. The transient solver settings were a second-order implicit transient formulation with a time step of 2 × 10^–3^ s.

### CFD model assessment

In order to compare the segment-to-segment number of ^90^Y-microspheres predicted by the simulation vs. the actual treatment, the mean activity per volume for each segment was obtained by the CFD simulation and the ^90^Y PET/CT.

For the CFD simulation, the number of microspheres that reached each segment was calculated considering the number of microspheres contained in a same-day calibration 3 GBq vial (44.48 ± 2.6 millions)^23^ and that the activity that reaches one segment in the simulations is the sum of the activity that leaves the truncated geometry through the outlets that irrigate that segment. With an average activity by microsphere of 50 Bq, the mean activity concentration for a selected segment (Bq/ml) was then estimated in order to compare with the data obtained from the ^90^Y PET/CT scan. The relative position between the hepatic artery branch outlets and tumors and segments of the liver was provided by MeVis files.

Using a dedicated voxel-based dosimetry software (PLANET Dose, DOSIsoft SA, Paris, France), ^90^Y PET/CT was anatomically co-registered to the pCT using an automated rigid registration method. This was manually corrected in case of matching errors assessed by visual inspection. Segmented liver MeVis DICOM files (the same as the ones used in the CFD model) obtained from the pCT were fused in the co-registered ^90^Y PET-pCT images study. The mean activity per volume (Bq/ml) of each liver segment (including tumor and healthy parenchyma) was obtained.

The results from the CFD simulation and ^90^Y PET/CT were compared in three patients treated with RE. Patients will be referred to as Patients 1, 2 and 3 and the different tumors from each patient will be designated as a, b, c, etc. Patient, tumor and treatment characteristics are summarized in Table 2.

**Table 2:** Information of the tumors and the RE procedures in each of the three patients.

Patient	Tumor name	Tumor volume (ml)	Activity (GBq)	*k*_2,*s*_ (ml/min/100 ml)^a^	k*_1,s_ (ml/min/100 ml)^b^	Segment location by MeVIS	Injection velocity (m/s)	Comments
Patient 1	1a	11.64	0.19	54.7	6.9	Segment 2	0.684	Two injections for tumor 1a and one injection for tumors 1b, 1c, 1d, and 1e
			0.19			Segment 3	0.778	
	1b	2.81	0.8	40.2	6.2	Segment 8	2.01	
	1c	2.67		39.3	4.2	Segments 7 and 8	2.01	
	1d	6.22		38.3	2.3	Segment 7	2.01	
	1e	15.65		41.4	5.0	Segment 7	2.01	
Patient 2	2a	2.53	0.50	36.7	3.1	Segment 7	0.52	One injection per tumor
	2b	4.47	0.20	42.7	5.2	Segment 8	0.45	
Patient 3	3a	63.87	0.82	96.7	12.0	Segments 5, 6 and 7	1.85	One injection for the three tumors
	3b	8.92		129.4	11.4	Segment 8	1.85	
	3c	6.21		103.4	11.9	Segments 7 and 8	1.85	

^a^Tumoral arterial perfusion.

^b^Healthy parenchyma arterial perfusion.

#### Patient 1

**Figure 2:**
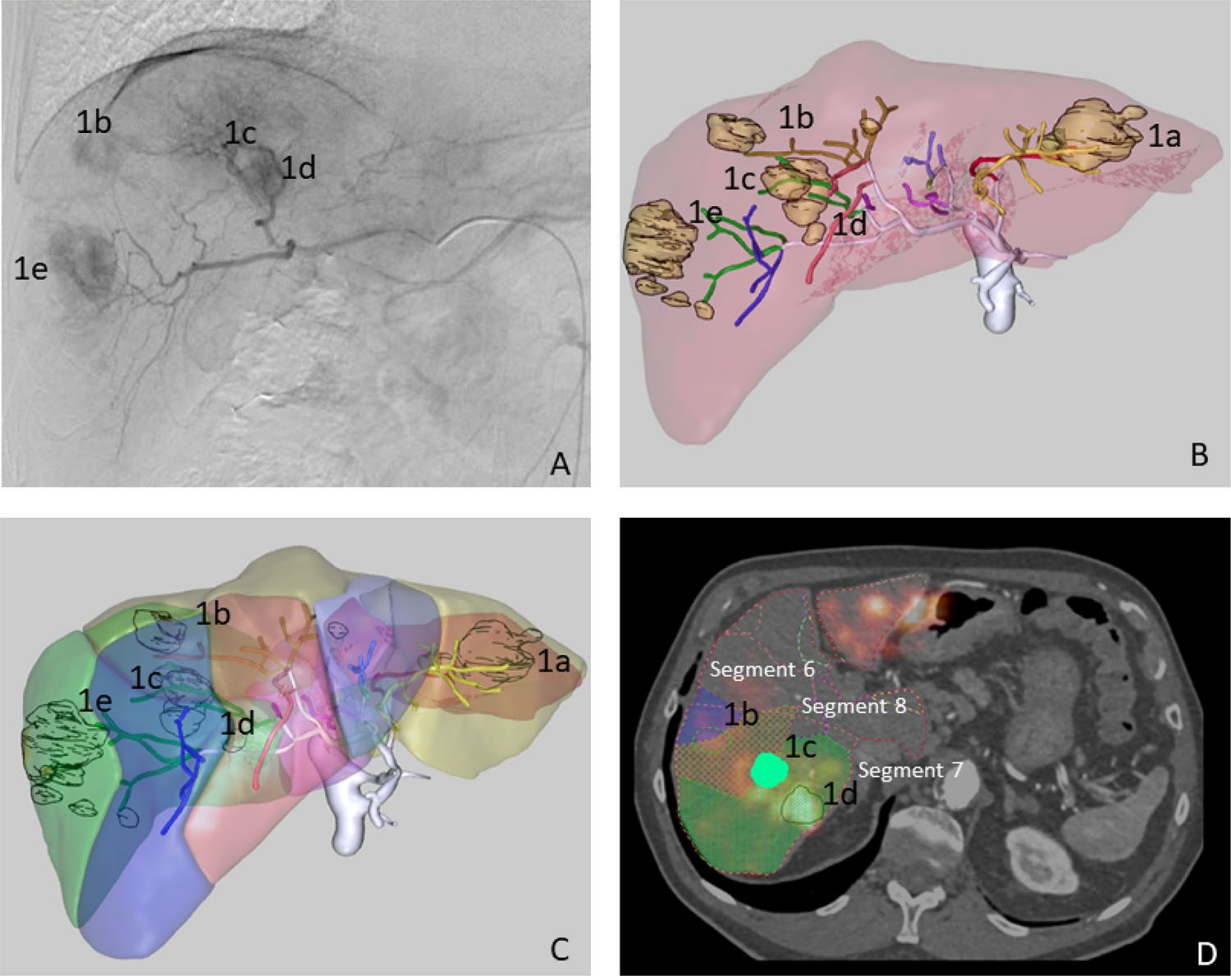
Patient 1. Multinodular HCC in the arteriography study (**A**). MeVis study showing arterial and liver segment territories (**B**,**C**) as well as tumor nodules (1a to 1e). MeVis liver segment contours imported on co-registered ^90^Y PET-pCT images (**D**) showing that tumor 1b was mainly irrigated by segment 8 artery (and slightly by segment 6 artery); 1c by segment 7 and 8 segment arteries; and 1d exclusively by segment 7 artery.

Patient 1 had a multinodular (5 nodules) HCC involving both liver lobes. The calculated Hepatopulmonary Shunt (HPS) was 6.4%. During angiographic mapping evaluation, an accessory left gastric artery was embolized to avoid the undesired gastric deposition of microspheres. Three microsphere infusions were performed through segment 2 (0.19 GBq) and segment 3 (0.19 GBq) arteries and the right hepatic artery (0.8 GBq). A Direxion Transend-14 System’s microcatheter (Boston Scientific, Watertown, MA, USA) with a curved end, was used during the procedure. The right hepatic artery injection allowed all tumors in the right lobe to be reached (tumors 1b, 1c, 1d and 1f.); while segment 2 and 3 artery injections covered a tumor located between both segments (1a). (see Fig. 2).

#### Patient 2

**Figure 3:**
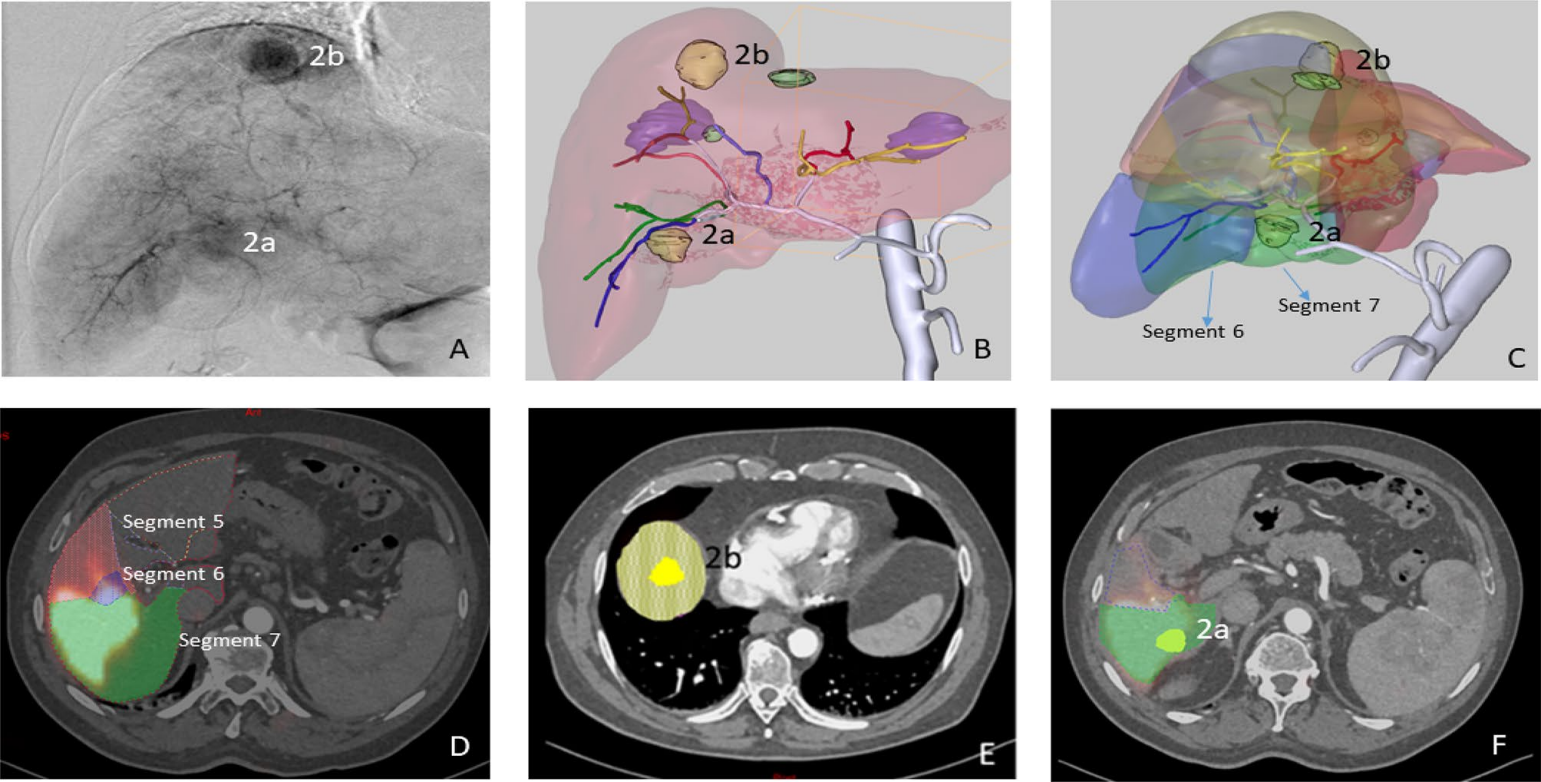
Patient 2. HCC with two hypervascular nodules in the arteriography study (**A**). MeVis study showing the arterial and liver segment territories (**B**,**C**) as well as the tumoral nodules (2a irrigated only by segment 7 artery and 2b only by segment 8 artery). Since the treatment was administered through 5–6 and 7–8 segment arteries, ^90^Y-microspheres deposition was seen in segments 5, 6, 7 (**D**,**F**) and 8 (**E**) in the MeVis liver segmentation fused on co-registered PET-pCT images.

Patient 2 had a multinodular (2 nodules) HCC with one nodule in the liver dome and another in segment 6. The calculated HPS was 5%. Anatomically normal hepatic vasculature and a partial thrombosis of the main portal vein were observed during the angiographic procedure. Microspheres were injected through segments 5–6 (0.5 GBq) and 7–8 (0.2 GBq) arteries for the total coverage of both lesions (see Fig. 3). A Progreat MC-PP27131 (Terumo Medical Corporation, Somerset, NJ, USA) microcatheter was used.

#### Patient 3

**Figure 4:**
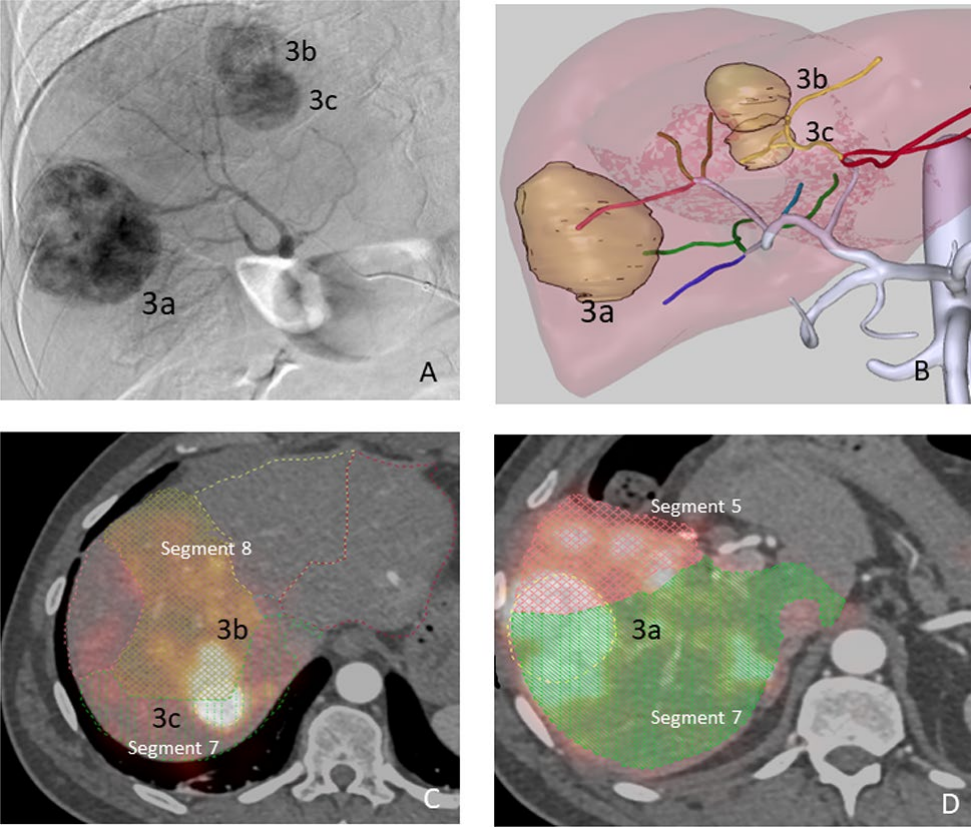
Patient 3. Multinodular HCC in the arteriography study (**A**). MeVis study showing arterial liver segment territories (**B**) as well as tumor nodules (3a to 3c). Using the imported MeVis liver segment contours on co-registered ^90^Y PET-angioCT (E and D), it is posible to define that tumor 3b is feeded by segment 8 artery, 3C by segment 7 and 8 arteries and tumor 3a by segment 5 and 7 arteries.

Patient 3 presented a multinodular (3 nodules) HCC, with nodules located in segments 8, 7–8 and 5–7 (see Fig. 4). The calculated HPS was 3.5%. No anatomical variants were observed during the angiographic procedure. In this case, a single injection (0.82 GBq) was performed through the right hepatic artery. A Progreat MC-PP27131 (Terumo Medical Corporation, Somerset, NJ, USA) microcatheter was used.

### Statistical analysis

Statistical analysis was performed using MedCalc Version 19.6.1. Correlation between variables was tested using the Spearman´s rank correlation coefficient (r_s_). Bland–Altman bias plot was used to analyze the differences (mean) between activity distribution values (%) obtained with CFD and with ^90^Y PET/CT and also between the predicted blood flow distribution and the actual activity distribution measured in ^90^Y PET/CT; limits of agreement (LoA) were calculated as the mean of differences ± [1.96·standard deviation]^24^; the 95% confidence interval of the LoA was also obtained.

## Results

Figure 1 shows some the CFD details for patients 1, 2 and 3 together with an image of the computational domain and the angiography on which it is based.

### Patient 1

**Figure 5:**
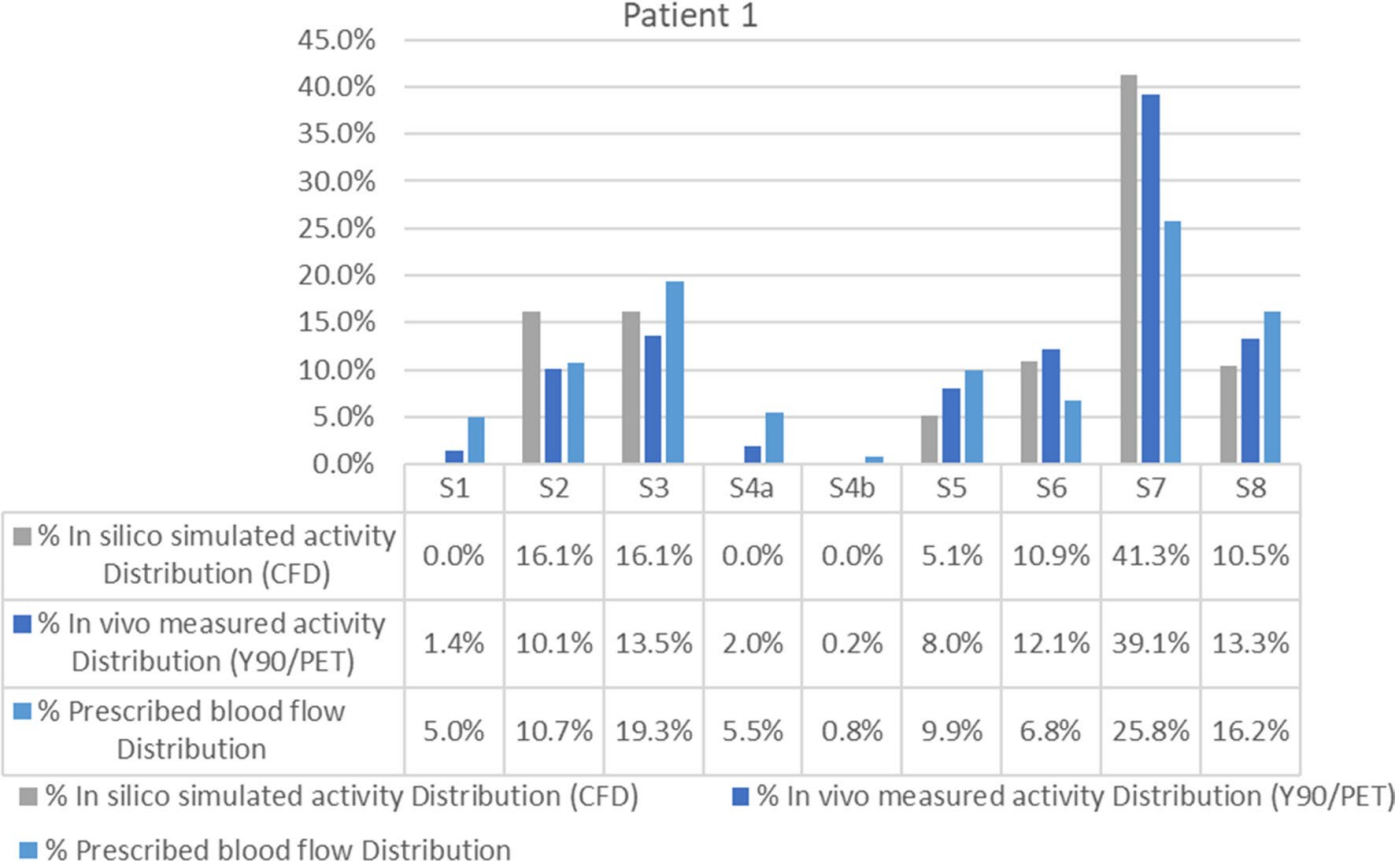
Segment-to-segment calculated activity distribution (CFD), measured activity distribution (^90^Y/PET), and prescribed blood flow split for Patient 1.

For this patient, the percentage point difference between ^90^Y PET/CT activity and CFD prediction [that is defined as |% Simulated distribution (CFD) - % Measured distribution(^90^Y PET/CT)|] was < 3 in all segments except in segment 2, which showed a difference of 6 percentage points (10.1% vs. 16.1%). The average difference between the distribution predicted by CFD and the actual distribution measured in the ^90^Y PET/CT was 2.36 percentage points (Fig. 5). The blood flow distribution presented higher differences with the CFD simulation (5.6 percentage points) and with ^90^Y PET/CT (4.16 percentage points) in the same segments.

### Patient 2

**Figure 6:**
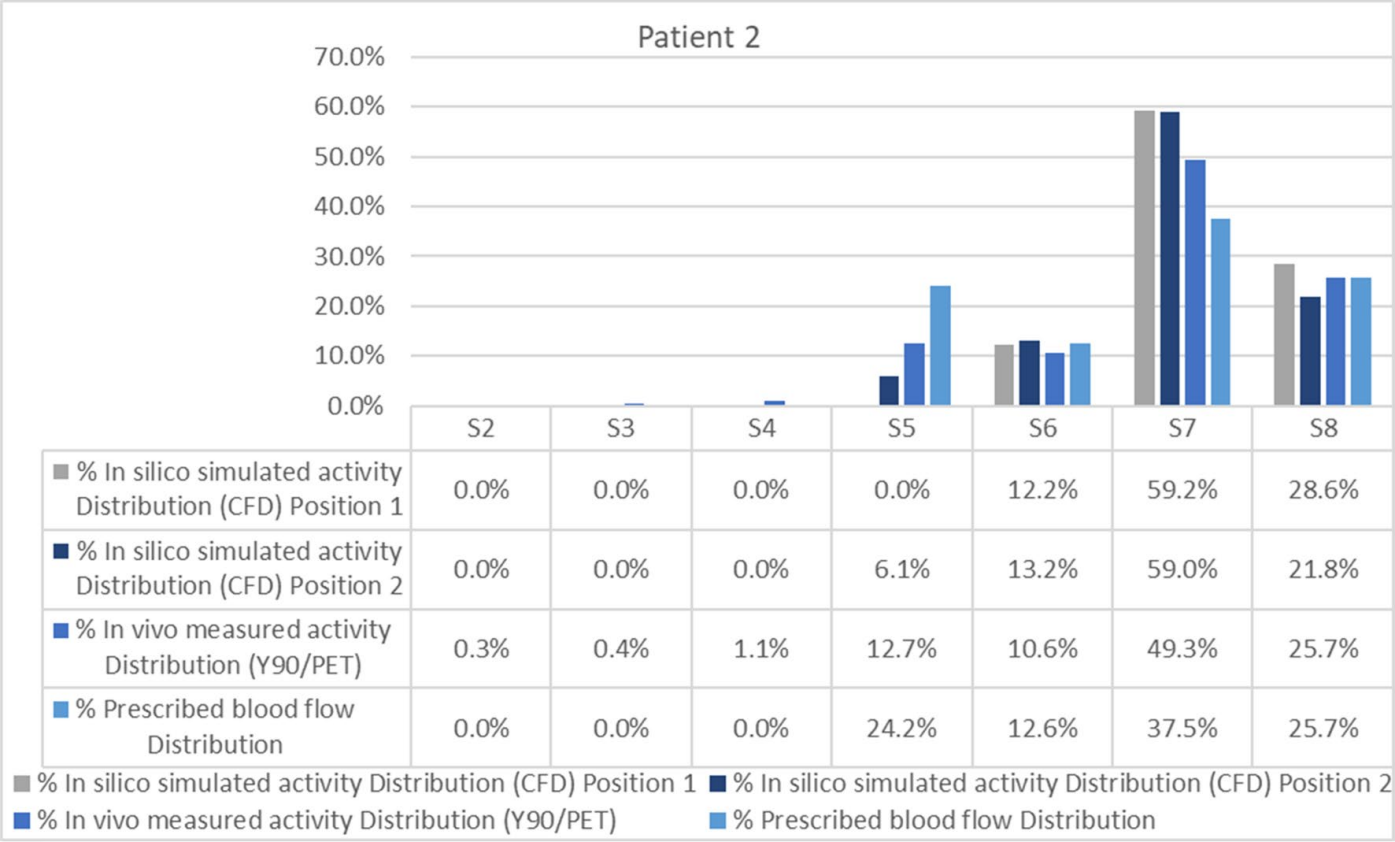
Segment-to-segment calculated activity distribution (CFD), measured activity distribution (^90^Y/PET), and prescribed blood flow split for Patient 2.

The average difference between the activity distribution predicted by the simulation and that measured by the ^90^Y PET/CT was 4.12 percentage points (Fig. 6).This difference was mainly due to discrepancies in one segment. The CFD model, developed according to the catheter position defined in the angiographic image captured during RE, predicted no deposition of ^90^Y-microspheres in segment 5. However, the PET/CT (Fig. 3) showed that ^90^Y-micropsheres did reach segment 5. The proximity of the catheter tip to an arterial bifurcation and the misinterpretation of the precise location of the tip may have been the cause of this discrepancy. As a consequence, the simulation was repeated after placing the microcatheter tip just before the bifurcation. The difference obtained with the new simulation was 3.51 percentage points (see position 2 in Figs. 1 and 6).

In this patient the blood flow distribution showed an average difference of 3.87 percentage points with the ^90^Y-microspheres deposition.

### Patient 3

**Figure 7:**
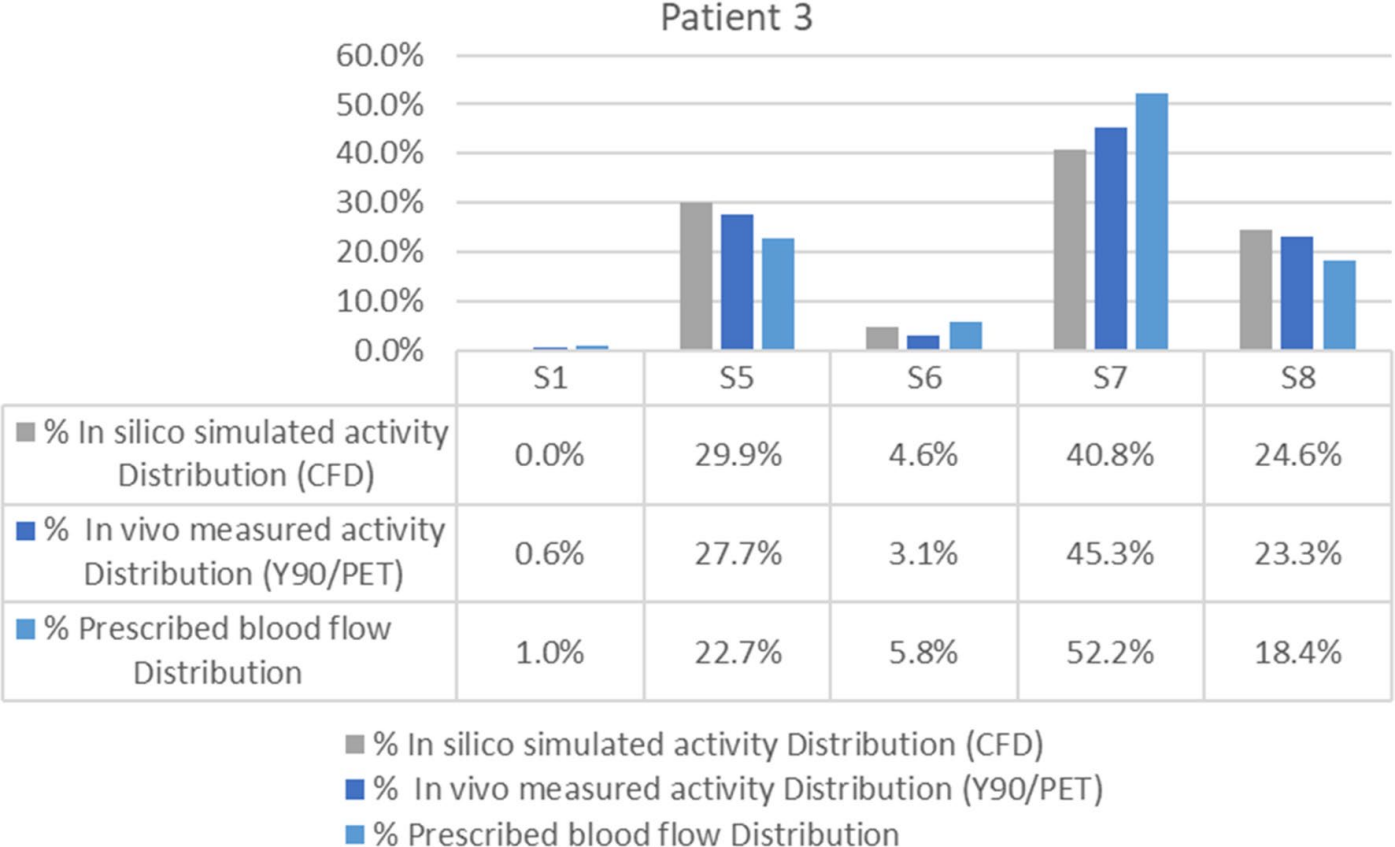
Segment-to-segment calculated activity distribution (CFD), measured activity distribution (^90^Y/PET), and prescribed blood flow split for Patient 3.

The average difference between the activity distribution according to the CFD simulation and that measured by the ^90^Y PET/CT was 2.02 percentage points (see Fig. 7). In this patient, the blood flow distribution showed an average difference with ^90^Y PET/CT activity of 5.4 percentage points and of 4 with CFD simulation.

**Figure 8:**
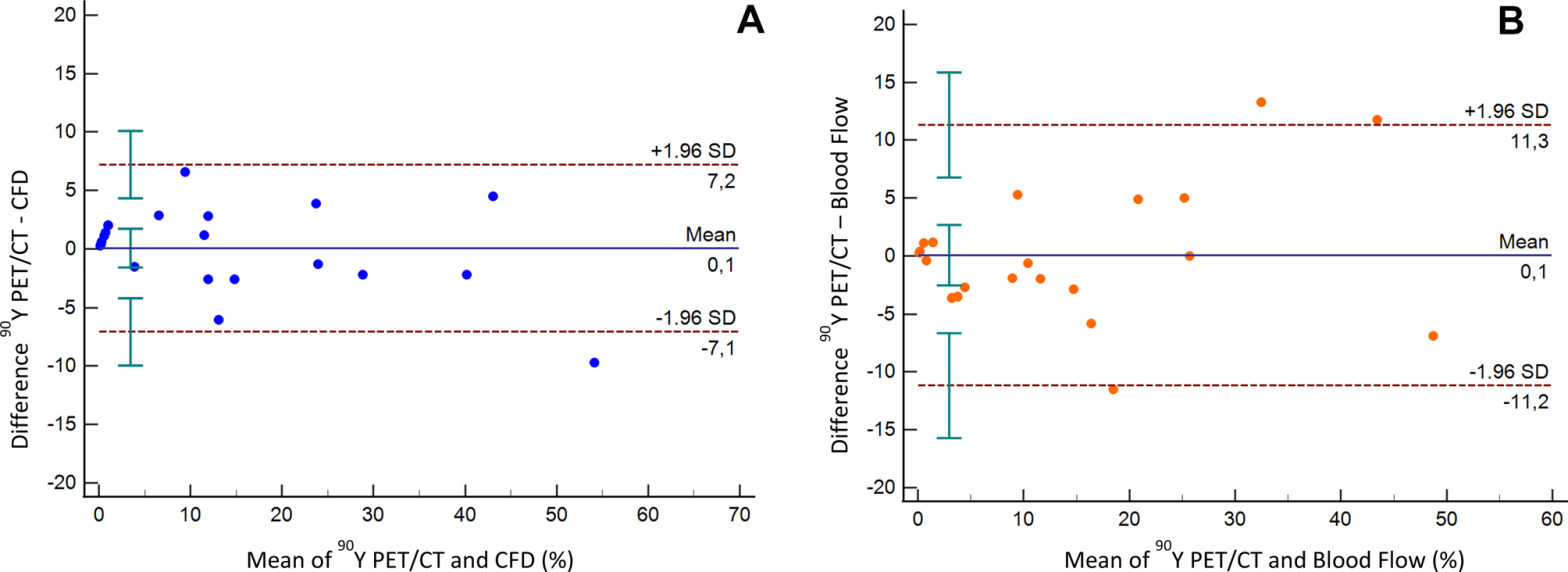
Bland–Altman plots for the differences in distribution in the 21 liver segments studied. (**A**) Difference between in vivo (^90^Y PET/CT) measured activity distribution and in silico (CFD) simulated activity distribution (%). (**B**) Difference between in vivo (^90^Y PET/CT) measured activity distribution and prescribed blood flow distribution (%). Solid blue lines indicate the mean of differences, dotted lines indicate upper and lower limits of agreement (LoA) and error bars the 95% confidence interval for both the upper and lower LoA and the mean difference.

Spearman´s coefficient analysis showed a strong correlation (r_s_ = 0.951) between the activity distribution (%) predicted by CFD and that obtained with ^90^Y PET/CT for all patients (*p* ≤ 0.001). Likewise, the analysis of Bland–Altman plot of the 21 segments (Fig. 8) revealed a very good agreement between CFD and ^90^Y PET/CT activity distribution (mean difference = 0.1%; LoA of the differences = − 7.1% and 7.2%; confidence intervals of the 95% LoA = − 9.9 to − 4.2% and 4.3 to 10.1%). The prescribed blood flow distribution (%) also demonstrated a strong correlation with ^90^Y PET/CT activity distribution (r_s_ = 0.96; *p* ≤ 0.001). Nonetheless, Bland–Altman plot analysis showed a wider LoA than for CFD and ^90^Y PET/CT (mean difference = 0.1%; LoA of the differences = − 11.2% and 11.3%; confidence intervals of the 95% LoA = − 15.7 to − 6.6% and 6.8 to 15.8%).

## Discussion

In this study, a patient-specific CFD model of the hemodynamics of the hepatic artery during RE has been compared with in vivo data obtained from ^90^Y PET/CT post-RE. To the best of our knowledge, this kind of in vivo validation has not been reported before.

The CFD simulated segment-to-segment ^90^Y-microsphere distribution was compared with the measured segment-to-segment activity distribution in ^90^Y PET/CT. The results obtained in three patients and six different simulations (one for each infusion) showed a high level of agreement, with an average percentage points of 2.36 and 2.02 for two patients. One patient (Patient 2) presented a higher discrepancy, due to a misinterpretation of the exact position of the catheter tip, further aggravated by its proximity to an arterial bifurcation. As previously shown, minimal changes in the position of a catheter when it is close to an arterial bifurcation can cause dramatic changes in the results obtained by the simulation. The exact radial position is not known, and this is a limitation and a source of error. Furthermore, as happened in this case, if the artery has a curved shape proximal to the bifurcation, the microcatheter may tend to be "eccentric" and the microspheres would be released not in the center but close to the vascular wall. Although a new simulation was modeled and the agreement obtained was improved, it still showed worse results than in the other two patients. These results highlight the importance of the careful location of the catheter when it is in the proximity of a bifurcation with the aforementioned characteristics and the advisability of using curved microcatheters for a better adaptation to the shape of the artery.

An important aspect of the study refers to ^90^Y-microsphere distribution per segment. Overall, both the CFD model and the prescribed blood flow results showed a good agreement with the actual distribution in ^90^Y PET/CT. However, as can be seen in the segment-to-segment distribution figures and in the Bland–Altman plot, the latter was relatively different to the blood flow distribution on several occasions. This indicates that particle distribution is influenced, but not determined, by blood flow. This claim is supported by other studies in which blood flow distribution was also studied^8,11,13^. ^90^Y-microsphere deposition is also affected by local parameters such as the distance between the catheter tip and an arterial bifurcation, the ratio of microsphere velocity to blood flow velocity, the type of catheter tip, and the radial position of the catheter in the artery^8^. This gives rise to two considerations. On the one hand, that all these parameters can be optimized for a better patient-specific treatment design using CFD models. The aforementioned local factors can be numerically analyzed and provide some useful information to the interventional radiologist about the most effective injection location, type of device, and injection velocity. The methodology presented here could pave the way toward personalized RE planning supported by numerical simulations from a CFD model of the patient-specific hemodynamics during RE. On the other hand, since all these local parameters are referred to as modifications in the 3D space, adoption of a 3D approach in the development of the CFD model, as used in this study, seems unquestionable. Not using a complete 3D approach or Bcs based on perfusions are the main limitations affecting some previous studies investigating the use of computational models to predict microsphere distribution after RE^25–27^.

In order to reduce the time spent on the simulation, only four cardiac cycles were modeled. The simulation of four cardiac cycles requires about 12 h on a 120-core workstation. This means that for the simulation of a whole RE procedure several weeks or months would be necessary. Whether microsphere distribution in the outlets of the computational domain during the first seconds of the injection is representative of the whole procedure, is an unanswered question. Moreover, this model does not take into consideration the occlusive effect that the consecutive deposition of the microspheres can have in small vessels or the possible alteration of the vascular wall by progressive handling during the procedure. Although there is indeed an occlusive effect inside the segments (outside the computational domain), it is hypothesized that the occlusive effect does not influence the blood flow along the first branches which are those included in the computational domain. It is important to state that the microsphere concentration in the vial decreases exponentially with time, meaning that most microspheres are infused during the first phase of the treatment. The occlusive effect will be present in the late stages but, at that time, most of the microspheres are already inside the segments. In this study, the focus is on the percentage of activity that goes to each segment and not in the activity distribution inside the segment. However, and in view of the results obtained, our hypothesis is not far from the reality and the simulation of just four cycles allows the final distribution of the ^90^Y-microspheres to be adequately predicted.

A personalized multidisciplinary strategy for RE would allow those patients who can benefit the most from the treatment to be selected, defining the adequate indication (curative, neoadjuvant to surgical or palliative); the best strategy depending on the tumor type, lesion location and the vascular anatomy; the optimal infusion point; and the use of tailored dosimetry for an effective but safe procedure. In this setting, CFD simulations performed before the conventional planning with ^99m^Tc-MAA could also complement it, determining the appropriate injection characteristics to effectively direct the ^90^Y-microspheres. Once this methodology has been validated, the next step would be the development of a user-friendly platform for the personalization of RE. This would only require a pCT to obtain the arterial liver segmentation and calculate segmental perfusion. With some improvements to the CFD model implemented in this study (such as removing from the computational domain the arterial branches that are not essential in determining the segment-to-segment microsphere distribution or reducing the order of the governing equations to 1D or even 0D equations in some branches), the results of the simulation could be obtained within a few hours. Nevertheless, before considering the implementation of the CFD model in RE planning, the inverse validation must be performed: to direct the injection point and velocity of ^90^Y-microspheres using the CFD model simulation and to confirm that the distribution of microspheres is as expected.

This study presents a number of limitations. Firstly, it includes a small number of patients from a single institution. Nevertheless, although we believe that six ^90^Y-microspheres injections and simulations provide sufficient information to validate the approach, prospective studies with a higher number of patients are warranted. Secondly, as mentioned above, the model does not consider whether a certain degree of stasis occurs during the RE procedure and the hemodynamic changes that it produces. However, stasis usually occurs at the final stages of the procedure, and by that time, a high proportion of microspheres has already been introduced. Moreover, the possible influence that the presence of the catheter or the microspheres inside the vessels can have on the precise estimation of the segment perfusion with the pCT scan, and the impact on flow obstruction induced by the catheter, were not evaluated.

## Conclusion

The distribution of ^90^Y-microspheres after injection into a hepatic artery is governed not only by the flow distribution but also by other 3D local factors. We have developed a 3D computational model that includes pre-treatment patient-specific data but also local factors that can affect microsphere distribution. The capability of this model to predict the ^90^Y-microspheres distribution has been validated in vivo, showing that CFD models could be used to personalize RE treatments.

## Data Availability

The datasets generated during and/or analysed during the current study are available from the corresponding author on reasonable request.
